# Carotid artery plaque assessment using CT with iodine mapping

**DOI:** 10.1007/s00330-023-10276-0

**Published:** 2023-10-06

**Authors:** Michimasa Suzuki, Yusuke Onozato, Jiro Kondo, Shinsuke Kyogoku

**Affiliations:** 1 Department of Radiology, Juntendo University, Uraysu Hospital, Chiba, Japan; 2 Faculty of Health Science, Juntendo University, Tokyo, Japan

Vessel wall imaging of the carotid artery is a very important tool for assessing stroke risk and planning treatment strategies [1, 2]. Until recently, a method for evaluating plaque composition by Vessel wall imaging using multi-contrast MRI has been established. However, there is a problem with this method in terms of the length of imaging time and a protocol has been put in place to add an imaging test at the end, further lengthening this imaging interval. In addition, specific contraindications stemming from MRI examinations and contrast agents have hampered examination [3]. For this reason, patients have been asked to endure nearly an hour of scanning. A drawback of this examination is that the scanning range is short, and how to collect a large amount of blood vessel wall information in a limited amount of time has been an urgent issue in this field.

CT examination of the carotid artery has the advantages of high resolution and ease of access and is widely used for stenosis evaluation. It is very effective in evaluating calcification distribution before carotid artery stent treatment. Previous studies have shown that delayed-phase CT imaging provides a great deal of information regarding plaque [4]. In CT, the difference between a lesion and a non-lesion area, and the difference in properties within the lesion are expressed by the contrast of the difference in the CT value. In recent years, techniques for enhancing contrast have been explored. In particular, for lesions with intraplaque hemorrhage, which are regarded as fragile plaques, it is necessary to be able to determine the fine enhancement effect of neovascularization [2], and a fine contrast is required. For that purpose, only the lesions to be imaged need to be clearly and strongly delineated. Under such circumstances, one solution is to create an image in which the iodine contained in the contrast agent is emphasized, that is, an iodine image. There is a method using dual-energy and a method using single energy to obtain an iodine mapping image. Since the dual-energy method requires only one scan time, it has advantages such as a reduction in the amount of contrast agent used, beam hardening correction, and image adjustment after imaging [5]. However, equipment is indispensable, and the imaging method and imaging range are limited. On the other hand, the single energy method has the advantage of not depending on the model. Subtraction Iodine Mapping can be acquired without requiring a special imaging model. However, this requires registration using advanced non-linear (registration) algorithms optimized for soft tissues. With the development of registration technology, which is susceptible to body movements, this technique has already been clinically applied to the abdominal region [6]. Theoretically, iodine mapping from contrast-enhanced and non-contrast subtraction acquired at the same voltage can be useful because it is more likely to yield tissue CT differences than those acquired at different voltages. A contrast-enhanced image (CE Boost image) is created by adding the denoised image of this iodine map to the delayed contrast-enhanced image [6], and by using the iodine maps, BBCT is generated to clarify the plaque burden.

In this issue of *European Radiology*, Lu et al. [7] evaluate carotid artery plaque using BB-CT. Using vessel wall magnetic resonance images (VW-MR) as the gold standard for evaluating plaque components, BBCT was shown to be more accurate in evaluating low-attenuation plaque, mixed components, and plaque volume than CTA. A comparison of the performance of BBCT in evaluating stenosis rate was also performed, and it was shown that detection was more sensitive than CTA.

The enhancement of contrast using subtraction improves the ability to distinguish contrast-enhanced and non-enhanced regions and contributes to the qualitative evaluation of all sites. These results indicate that BBCT can become a one-stop shopping tool for carotid artery evaluation, including stenosis rate and plaque evaluation, without increasing additional radiation exposure or contrast agents during a limited time in daily clinical practice. It is an intriguing field and it would not be surprising if a new method of evaluation of carotid artery plaques by black-blood CT was proposed.

## Declarations

### Guarantor

The scientific guarantor of this publication is Michimasa Suzuki, Chief of the Department of Radiology, Juntendo Urayasu Hospital.

### Statistics and biometry

No complex statistical methods were necessary for this paper.

### Methodology

• commentary
